# Revolutionizing Dental Polymers: The Versatility and Future Potential of Polyetheretherketone in Restorative Dentistry

**DOI:** 10.3390/polym17010080

**Published:** 2024-12-31

**Authors:** Noha Taymour, Ahmed Abd El-Fattah, Sherif Kandil, Amal E. Fahmy, Naif H. Al-Qahtani, Abdulrahman Khaled, Yousif A. Al-Dulaijan, Mohamed Abdel-Hady Gepreel

**Affiliations:** 1 Department of Substitutive Dental Sciences, College of Dentistry, Imam Abdulrahman Bin Faisal University, P.O. Box 1982, Dammam 31441, Saudi Arabia; ntyoussef@iau.edu.sa; 2 Department of Materials Science, Institute of Graduate Studies and Research, Alexandria University, El-Shatby, Alexandria 21526, Egypt or a_abdelfattah@alexu.edu.eg (A.A.E.-F.); s.kandil@usa.net (S.K.); 3 Department of Chemistry, College of Science, University of Bahrain, Sakhir P.O. Box 32038, Bahrain; 4 Department of Dental Materials, Faculty of Dentistry, Alexandria University, Azarita, Alexandria 21526, Egypt; amal_ezzeldin99@yahoo.com; 5 College of Dentistry, Imam Abdulrahman Bin Faisal University, P.O. Box 1982, Dammam 31441, Saudi Arabia; 2210000179@iau.edu.sa; 6 Department of Materials Science and Engineering, Egypt-Japan University of Science and Technology (E-JUST), New Borg El-Arab City 21934, Egypt; geprell@yahoo.com

**Keywords:** Polyetheretherketone (PEEK), implants, prosthesis, thermoplastics, tissue regeneration, bone regeneration

## Abstract

Polyetheretherketone (PEEK) has emerged as a revolutionary material in modern dentistry because of its unique combination of mechanical strength, biocompatibility, and versatility. This literature review examines the current applications and future potential of PEEK in various dental disciplines. PEEK’s favorable properties, including its low specific weight, high strength-to-weight ratio, and ability to be easily machined, have led to its adoption in prosthetics, implantology, and dental esthetic restorations. This material has shown promise for fabricating crowns, bridges, removable partial denture frameworks, and implant components. PEEK’s radiolucency and bone-like elastic modulus make it particularly suitable for dental implants and abutments. Additionally, its resistance to degradation and compatibility with various surface treatments enhances its long-term performance in the oral environment. While challenges such as bonding to other dental materials and aesthetic limitations exist, ongoing research is addressing these issues through surface modifications and composite formulations. As the dental field continues to evolve, PEEK’s adaptability and biocompatibility position it a key player in the development of next-generation dental materials and techniques, potentially transforming patient care and treatment outcomes in dentistry.

## 1. Introduction

**Figure 1:**
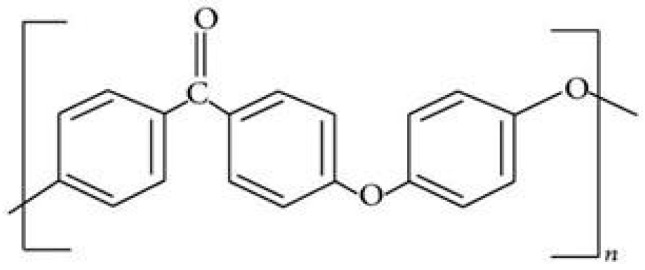
Chemical structure of PEEK [1].

PEEK (Polyether ether ketone) is a semi-crystalline, high-performance engineering thermoplastic material. It is a special polymer material composed of repeated units of one ketone bond and two ether bonds in the backbone of the polymer, as shown in Figure 1 [1]. PEEK polymers are obtained by step-growth polymerization by the dialkylation of bisphenolate salts. The reaction is conducted at around 300 °C in polar aprotic solvents, such as diphenyl sulfone [2]. PEEK’s unique properties enable its use in diverse fields, including biomedical, aerospace, automotive, and electrical industries. It demonstrates exceptional resistance to fatigue, creep, chemical degradation, and wear, making it particularly valuable for high-performance applications. These characteristics have led to PEEK’s widespread adoption in structural components, high-temperature environments, and advanced engineering solutions [3]. PEEK’s high resistance to oxidative deterioration and its strength-to-weight profile are high enough to compete with metals like aluminum [4]. Regarding thermal properties, PEEK is highly temperature-resistant, with a glass transition temperature of around 143 °C (289 °F) and a melting point of around 343 °C (662 °F) [5]. In addition, PEEK has excellent chemical resistance properties; it is chemically resistant to most organic and inorganic chemicals, as well as to hydrolysis, radiation, and sterilization [5]. PEEK is a highly durable material with excellent mechanical and chemical resistance properties that are retained at high temperatures. Its density is 1320 kg/m^3^, Young’s modulus is 3.6 GPa, and tensile strength is 90 to 100 MPa [6]. PEEK is readily machinable in the solid state, allowing the creation of complex shapes and designs [7].

There are several grades of PEEK, including both extremely pure PEEK and PEEK reinforced with glass, carbon fiber, or other technical plastics. In the industry, PEEK comes in four main grades: 30% carbon-filled PEEK, 30% glass-filled PEEK, bearing-grade PEEK, and unfilled PEEK [6]. Different processing techniques are used to enhance PEEK’s properties [8,9]. PEEK pellets are generally recommended for extrusion, monofilament, and wire coating operations, while PEEK powder is used for extrusion compounding when fillers are added to enhance PEEK properties, such as expanded graphite (EG) [10]. PEEK can be manufactured using conventional injection molding equipment. To achieve a semi-crystalline component that fully utilizes PEEK’s special set of characteristics, the mold temperature is crucial [11]. Compression molding is another process that produces PEEK with excellent mechanical properties; it is recommended for use with PEEK granules/rods. However, the key factors affecting the uniformity of compression-molded PEEK are the mold temperature, molding pressure, and ram speed (or the rate of application of pressure) [12]. Compression-molded PEEK samples showed higher crystallinity in comparison to injection-molded samples; subsequently, they were found to have higher strength compared to injection-molded PEEK samples [13]. Lee DJ et al. [14] have compared the mechanical properties (tensile, fatigue, and creep) of carbon fiber-filled PEEK using injection and compression molding techniques. Specimens with varying initial fiber lengths were created using both methods. Compression-molded specimens showed clear differences in mechanical properties based on fiber length, whereas injection-molded samples displayed consistent tensile and fatigue strengths regardless of the initial fiber length. Further research is needed to optimize the process parameters for specific applications and evaluate the long-term performance and success rates of compression-molded PEEK.

In addition to injection molding and compression molding, two other manufacturing methods have become increasingly significant for PEEK in dental applications. Subtractive manufacturing (CAD/CAM) has emerged as a primary technique for fabricating fixed and removable prosthodontics using PEEK [15]. This method encompasses milling and grinding processes to remove material from a PEEK block, shaping it into the desired form with high precision and accuracy, which are key factors for dental applications [4]. The resultant components exhibit smooth surfaces, often requiring minimal post-processing [16]. These features make subtractive manufacturing particularly suitable for creating fixed and removable prostheses that require precise anatomical fit and functional reliability [17]. 3D printing of PEEK offers several benefits for dental applications. Customization of implants and prostheses to patient-specific anatomy, reduced material waste compared to subtractive methods, ability to create complex geometries that may be challenging with other manufacturing methods, and the potential for faster production times for certain applications [18]. These manufacturing methods have significantly impacted the production of PEEK-based fixed and removable prosthodontics, offering dental professionals more options for creating precise patient-specific solutions [15].

CAD/CAM-milled PEEK remains the superior manufacturing method for dental applications demanding high mechanical strength and precision due to its homogeneous microstructure, which consistently delivers superior tensile, flexural, and fatigue strengths compared to 3D-printed PEEK [19]. While 3D printing of PEEK shows promise in customized and lower-load scenarios, it suffers from porosity and anisotropy unless optimized through parameters like infill rate, temperature, and post-print annealing [20,21]. Both methods offer similar baseline biocompatibility; however, the rougher surfaces of 3D-printed PEEK may promote higher osteoblast proliferation [15]. Surface treatments, such as sandblasting and plasma treatment, can enhance bioactivity for both methods, although uniformity and bacterial adhesion present challenges, especially for 3D-printed surfaces [15,19]. Clinically, milled PEEK is preferred for load-bearing applications like frameworks and implants due to its superior durability, while 3D-printed PEEK excels in customization for non-load-bearing or less demanding restorations, making it ideal for rapid prototyping [19,21]. Optimized parameters can improve the marginal fit of 3D-printed PEEK and rival milled PEEK in some cases. However, variability in printer settings, operator skills, and lack of standardized post-processing protocols limit the reliability of 3D-printed PEEK for clinical dentistry [22]. Advances such as carbon fiber-reinforced 3D-printed PEEK may help narrow the performance gap with milled PEEK, highlighting the need for further research and standardization [23].

**Figure 2:**
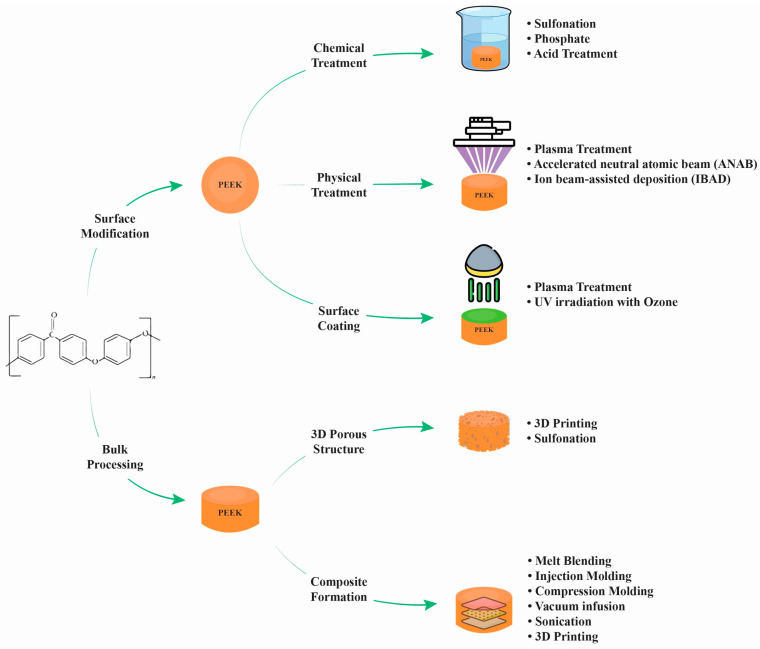
PEEK modifications and coating for enhanced bioactivity.

PEEK surface modification can be performed either by surface treatment alone or in conjunction with surface coating [24]. Surface treatments, both chemical and physical, can significantly increase PEEK’s bioactivity. For instance, PEEK surfaces’ chemical and physical characteristics have been modified via sulfonation and sub-millimeter laser machining, improving their bio-interaction [25]. Electron beam irradiation has also been used to modify the surface of PEEK, resulting in increased hydrophilicity and protein adsorption [26]. It has been observed that nitrogen plasma performs particularly well in PEEK implant applications. There has also been an investigation into further surface treatments, such as plasma ion implantation and nanomodification [27,28]. Rather than using physical treatments and composites, surface coatings have been employed to increase PEEK’s bioactivity [29,30]. For example, PEEK can be coated with bioactive substances such as hydroxyapatite or titanium to enhance its bioactivity and antibacterial properties [31,32]. The process of creating a nano-level surface topography by coating or mixing PEEK with nanoparticles has recently been the focus of a significant amount of research [33,34]. PEEK’s bioactivity can be significantly increased by surface treatment either by itself or in conjunction with surface coating. The use of surface coatings as substitutes for physical treatments and composites has been documented in the literature since the addition of bioactive particles to PEEK has raised questions about how to preserve the material’s mechanical properties. Figure 2 shows the different methods of PEEK processing for surface bioactivation.

**Figure 3:**
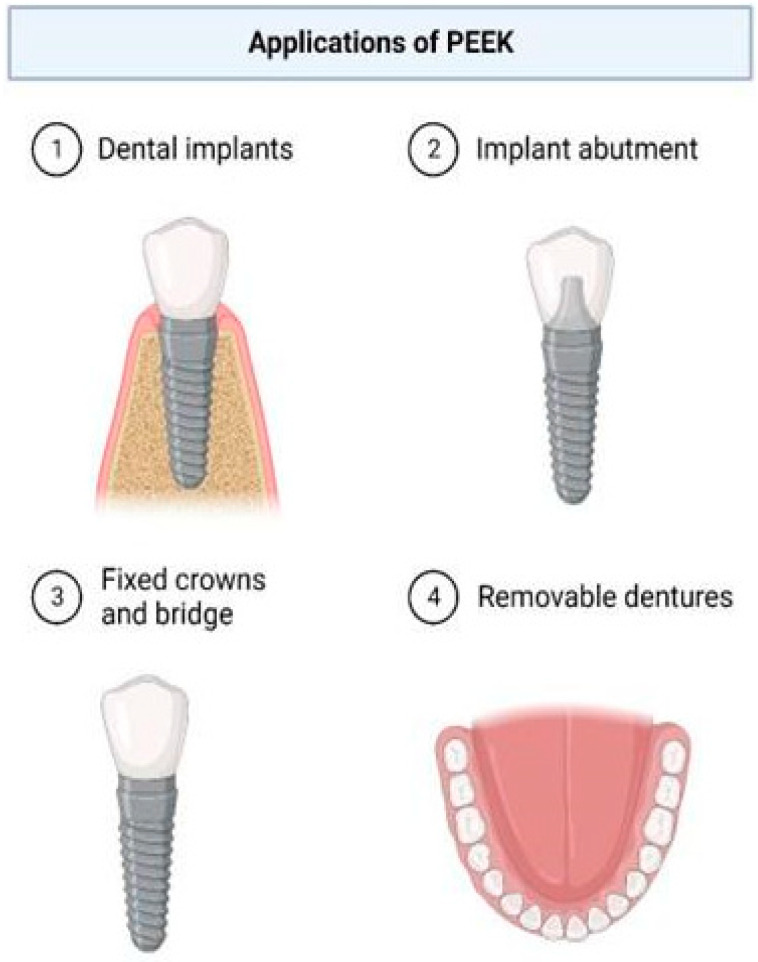
Common applications of PEEK in dentistry.

PEEK’s biocompatibility and ability to be blended with fibers and ceramics to enhance its mechanical properties and bioactive properties make it an ideal alternative for advanced medical and dental uses. As shown in Figure 3, PEEK is now utilized in crowns, posts and cores, maxillofacial prostheses, removable and fixed prostheses, dental implants, and implant abutments [35,36].

## 2. Methodology

### 2.1. Question Addressed by This Review

What are the different applications and benefits of PEEK in the field of restorative dentistry?

### 2.2. Literature Research

A narrative exploratory review was conducted. A literature search was carried out utilizing electronic databases such as PubMed (MEDLINE), Scopus, and Web of Science (WoS). Following that, a manual search of the literature was performed, including the reference lists of related and similar studies, to identify any new relevant research.

The main search terms were “PEEK”, “dentistry”, “implant”, “prosthesis” and “abutment”. Articles published between 2013 and 2024 were included. Any duplicate entries in the databases were identified and subsequently eliminated using Mendeley Reference Manager software (version 2.128) by Elsevier, based in Amsterdam, Netherlands.

Inclusion criteria encompassed peer-reviewed articles in English-written articles focusing on PEEK applications in the field of restorative dentistry, while exclusion criteria involved non-dental applications and non-English publications. Subtopics within PEEK in dentistry were identified and categorized based on the primary application areas, such as implants, prosthetics, and bone regeneration, as well as material properties, such as mechanical characteristics and biological activity. Papers were organized using a custom classification framework developed in Microsoft Excel, which allowed for efficient categorization and analysis.

### 2.3. Data Extraction

Data extraction was performed independently by two reviewers using a standardized form to collect information on the study design, sample size, key findings, and conclusions. Any discrepancies in data extraction were resolved through discussion to reach a consensus. The extracted data were then synthesized to identify trends, gaps in research, and potential future directions for PEEK applications in dentistry.

## 3. Results and Discussion

### 3.1. Advantages of PEEK over Traditional Materials

PEEK is a desirable material for various applications due to its exceptional mechanical, thermal, and chemical properties. PEEK has a higher strength-to-weight ratio, is more flexible, and is more biocompatible than traditional materials such as metals and ceramics [37].

According to Luo et al. [35], PEEK dental implants are more compatible with the mechanical characteristics of bone than metal and ceramic implants and show less stress shielding. Yongan et al. [38] focused on the improvement schemes for PEEK composites, aiming to enhance their mechanical properties, friction resistance, electrical conductivity, and thermal resistance. Tekin et al. [39] highlighted that PEEK’s low elasticity modulus can decrease stress-related issues and make it an effective replacement for current dental materials. The suitability of PEEK as a fixed partial denture framework was examined by Sinha et al. [40], who emphasized its superior mechanical properties and chemical resistance. JianBing et al. [41] mentioned that PEEK has prominent advantages in mechanical strength, thermal stability, and chemical stability. Researchers have suggested that PEEK offers superior properties and versatility compared to traditional materials in dental applications. Detailed comparisons between PEEK and other dental polymers are presented in Table 1.

#### 3.1.1. PEEK vs. Conventional Dental Alloy

PEEK material could be a viable alternative to conventional dental alloys. Tekin et al. [39] highlighted the good mechanical and electrical properties of PEEK, as well as its high biocompatibility, making it suitable for orthopedic and trauma cases. Schwitalla et al. [44] demonstrated that PEEK materials have a modulus of elasticity and strengths that meet the minimum requirements for dental applications. Additionally, Bathala et al. [36] discussed the advantages of PEEK, such as flexibility, radiolucency, thermal resistance, and biocompatibility, which make it a potential alternative to titanium and zirconia for dental implants. Researchers found that PEEK has the potential to be used in various areas of dentistry as a substitute for traditional dental alloys [45,46,47].

#### 3.1.2. PEEK vs. Dental Ceramics

PEEK and dental ceramics are contrasting materials in modern dentistry, each with distinct properties that influence their clinical applications. PEEK’s significant flexibility stands in stark contrast to the rigidity of dental ceramics [6]. This flexibility grants PEEK superior stress distribution and shock absorption capabilities, potentially reducing the risk of fractures and chips that often plague ceramic restorations [48]. Moreover, PEEK’s bone-like elasticity may contribute to better temporomandibular joint (TMJ) health by allowing more natural force distribution and adaptive load transfer [49,50]. While ceramics maintain their stronghold in aesthetically critical areas, PEEK’s unique combination of flexibility, fracture resistance, and biocompatibility has driven its increased adoption in prosthetics, implant components, and TMJ reconstruction [51,52]. As dentistry continues to evolve, the choice between PEEK and ceramics increasingly depends on balancing the mechanical properties, aesthetics, and patient-specific needs in each clinical scenario. According to Reddy et al. [53], PEEK dental implants better mimic the mechanical characteristics of bone and show less stress shielding. Elawadly et al. [54] evaluated the surface roughness and wettability of different PEEK specimens and suggested that filled PEEK materials exhibit favorable roughness and wettability properties, making them potential substrates for dental implants. Bekhiet et al. [55] investigated the wear and volumetric loss of PEEK against other dental materials and aimed to determine its efficacy as a permanent restoration.

### 3.2. PEEK in Dental Implantology

PEEK has been explored for various applications in dental implantology due to its unique properties. Since PEEK and bone have more similar mechanical characteristics, PEEK dental implants have been observed to have less stress shielding than titanium dental implants (Table 2) [6,56]. It is a promising material for dental implants due to its biocompatibility, mechanical strength, and resistance to wear and fatigue.

PEEK can be used as an alternative to traditional metal and ceramic materials for implant abutments [58]. PEEK abutments have been found to have a lower modulus of elasticity than titanium abutments, which can reduce the risk of implant failure due to stress shielding [6]. In addition, PEEK material radiolucency prevents interference with X-rays or other imaging techniques, which allows for better visualization of dental structures and implants during after-implantation diagnosis and treatment planning [59].

**Figure 4:**
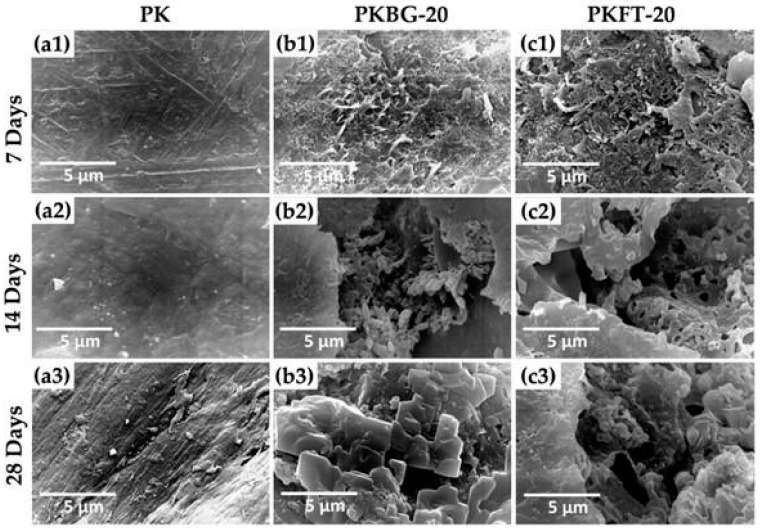
SEM images of (**a1**–**a3**) pure PEEK and (**b1**–**b3**) PEEK containing 20 wt.% 45S5 bioglass nanofillers, and (**c1**–**c3**) PEEK containing 20 wt.% forsterite (Mg_2_SiO_4_) nanofillers, demonstrating the apatite-formation capability following 7, 14, and 28 days of submersion in SBF. Pure PEEK (**a1**–**a3**) showed no changes on its surface. PEEK nanocomposites (**b1**–**b3**,**c1**–**c3**), however, encourage the production of apatite after immersion in SBF at all time points [6].

Nevertheless, PEEK is considered bioinert and does not readily bond with bone tissue, hindering its full integration with the surrounding bone following implantation. This hydrophobic nature arises from its aromatic ring and polyester functional groups, which prevent osseointegration and result in its separation from the bone [24,60]. The goal of recent research has been to increase the nanoscale bioactivity of PEEK implants in order to promote bone remodeling and osseointegration [24,61]. Comparing PEEK to bioglass-based PEEK nanocomposites, Taymour et al. [6] reported that PEEK with 20 wt.% forsterite in the nanocomposite formulation was the most efficient in PEEK’s bioactivation. This particular formulation greatly increased the material’s microhardness, flexural strength, and elastic modulus, in addition to enhancing PEEK’s bioactivation. This particular formulation greatly increased the material’s microhardness, flexural strength, and elastic modulus, in addition to enhancing PEEK’s bioactivation. Additionally, as illustrated in Figure 4 [6], both PEEK nanocomposites loaded with bioglass and those with forsterite nanofillers showed the potential to precipitate phosphate and calcium bone minerals on their surfaces during in vitro bioactivity evaluation using biomimetic simulated bodily fluid. Chayanun et al. [25] studied how sulfonation and sub-millimeter laser machining could improve the bioactivity of PEEK surfaces. The outcomes demonstrated that PEEK surfaces’ bioactivity was enhanced by the combination of these two methods. The classification of PEEK modifications and coatings for enhanced osseointegration is discussed in greater detail in the next section. However, further research is required to fully understand the potential of PEEK in implantology and improve its bioactivity without compromising its mechanical properties.

#### PEEK Modifications and Coatings for Enhanced Osseointegration

**Figure 5:**
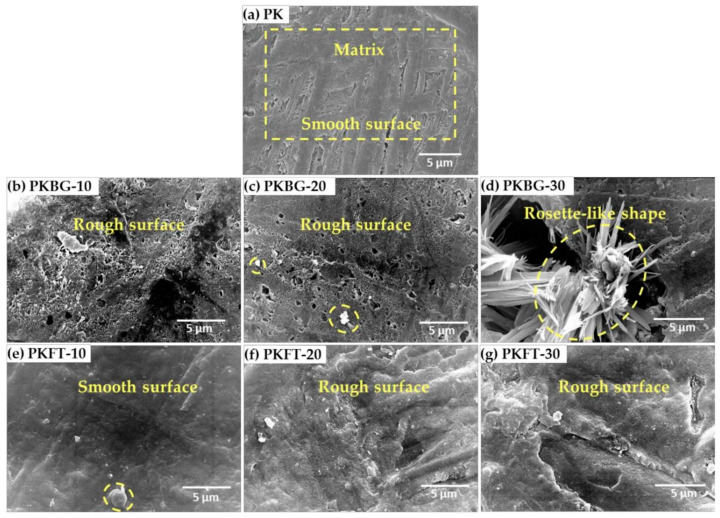
SEM images of pristine PEEK and PEEK loaded with 10,20,30 wt.% BG (45S5 bioglass) nanofillers and PEEK loaded with 10,20,30 wt.% FT (Mg_2_SiO_4_) nanofillers showing the morphologies of the surface displaying information about the rough and smooth areas: (**a**) PEEK’s smooth surface, (**b**,**c**) PKBG-10 and PKBG-20 rough surfaces, (**d**) rough bioglass nanoparticle aggregation on PKBG-30, (**e**) PKFT-10’s smooth surface, and (**f**,**g**) PKFT-20 and PKFT-30 low roughness with forsterite nanoparticles dispersed arbitrarily throughout the PEEK matrix. The average particle sizes of the BG and FT nanofillers are 40 ± 4 nm and 30 ± 5 nm, respectively [6].

**Figure 6:**
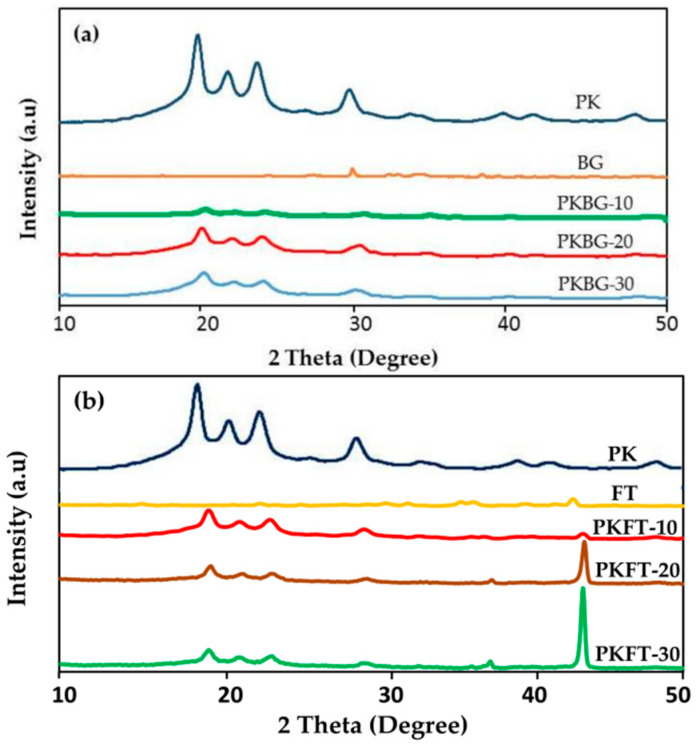
XRD patterns of (**a**) nanocomposites containing Bioglass (BG) nanoparticles and (**b**) nanocomposites including Forsterite (FT) nanoparticles, demonstrating how the addition of these particles affects the crystallinity of PEEK [6].

One of the major drawbacks of PEEK in dental implants is its bioinertness, as discussed above [6,27]. Surface modifications and blends can be applied to PEEK implants to enhance osseointegration. Three primary categories can be used to group these modifications as follows:

1. Bioactive agent surface functionalization: Bioactive agents can be introduced to the PEEK implant surface chemically or physically, as shown in Figure 2. Bioactive agents such as growth factors or peptides can promote cell adhesion, proliferation, and differentiation, leading to improved osseointegration [28]. Cyclic peptides, which have unique characteristics that promote the development of the endothelium, were bound to the PEEK surface by Young et al. [62] using ammonia plasma treatment. Furthermore, these conjugated cyclic peptides on the PEEK surface exhibit anticoagulant qualities by preventing platelet adhesion and activation, which may prevent blood clot formation. An et al. [63] created a dialdehyde cross-linked hyaluronic acid hydrogel coating on the surface of PEEK that was loaded with nerve growth factor (NGF) and platelet-rich plasma (PRP). These findings demonstrate that the hybrid hydrogel coating on the PEEK surface was capable of continuously releasing growth factors and exhibited good hydrophilicity. The hybrid hydrogel coating exhibited strong cell adhesion and facilitated the differentiation of MC3T3-E1 cells and angiogenesis of human umbilical vein endothelial cells (HUVECs), according to in vitro cell tests. Nevertheless, NGF did not enhance the hybrid hydrogel’s capacity for cell growth. Excellent osteogenic and angiogenic properties were also demonstrated by PEEK samples coated with the hybrid hydrogel in a rat tibial defect model. A comparatively green technique for grafting heparin onto PEEK through the thiol-ol reaction was developed by Goh et al. [64] by combining ozone and UV irradiation without the use of any chemical reagents or organic solvents. Instead, they used biocompatible cysteine, an amino acid, to thiolate heparin. In contrast to the rapid release of loaded bone morphogenetic protein-2 (BMP-2) from pure PEEK, heparin grafting on PEEK effectively immobilized BMP-2. Comparing heparin grafting to pure PEEK, the bioactivity of PEEK was improved in terms of MG-63 proliferation and osteogenic differentiation. It was only after heparin grafting that BMP-2 loading onto PEEK could have an impact on more specialized osteogenic activities such as alkaline phosphatase (ALP) activity and calcium deposition in MG-63.
2. Incorporation of PEEK with bioactive materials: Bioactive materials can be applied as surface coatings or incorporated into the PEEK structure as composites, which provide a favorable environment for cell attachment, proliferation, and differentiation, enhancing osseointegration [61]. Bioactive particles, such as hydroxyapatite (HA), strontium-containing hydroxyapatite, titanium dioxide (TiO2), tricalcium phosphate (TCP), bioactive glass, forsterite, and graphene, have been compounded with PEEK to increase the bioactivity and bone osseointegration of PEEK implants [6,65,66]. Silicate-based bioceramic-reinforced PEEK nanocomposites have been developed to improve the mechanical properties, bioactivity, and bone osseointegration of PEEK. PEEK/Bioceramics nanocomposites were fabricated using melt blending and compression molding techniques. The uniform dispersion of nanofiller particles within the PEEK matrix is crucial for improving the mechanical properties and bioactivity [9]. While surface treatments can offer some benefits in terms of improving the surface characteristics of PEEK, such as wettability and bioactivity, PEEK-based composites are designed to provide a comprehensive solution that addresses both mechanical strength and bioactive properties. These composites have shown promise in enhancing the performance of dental implants by promoting osseointegration and ensuring long-term implant stability [67]. PEEK composites offer a consistent distribution of bioactive fillers throughout the material, ensuring uniform bioactivity and mechanical performance across the entire implant, as shown in Figure 4. Surface treatments, on the other hand, may vary in effectiveness and may not provide uniform coverage or bioactivity throughout the PEEK surface. Researchers are tailoring the composition and content of bioactive fillers to optimize these properties. Surface treatments, on the other hand, may not provide the same degree of control over material properties and bioactivity [68]. In addition, PEEK-based composites typically incorporate reinforcing materials, such as hydroxyapatite (HA), glass fibers, or other bioactive fillers. These fillers can significantly enhance the mechanical properties of PEEK, including its tensile strength, elastic modulus, and microhardness. This improved mechanical strength can better withstand the stresses and loading conditions experienced in the oral environment, contributing to implant stability and longevity [69]. The mechanical properties of PEEK are dependent on the degree of crystallinity since higher crystallinity leads to higher tensile and flexural strengths. The degree of crystallinity is a positive factor in the mechanical properties of PEEK [70]. Pérez-Martín et al. [71] showed that carbon fiber-reinforced PEEK has improved mechanical properties with a higher crystallinity. However, Taymour et al. [6] found that by increasing the number of nanofillers by more than 20% by weight, despite the increased crystallinity, the mechanical properties were reduced, as shown in Table 3. The decline in the mechanical properties at higher filler content might be due to the fact that the higher amounts of nanofillers could restrict the mobility of the polymer chains, decreasing the degree of crystallinity, crystallite size, and crystallization growth rate, as shown in Figure 5 and Figure 6 and Table 3 [72].

3. Construction of three-dimensional porous structures: Three-dimensional porous structures can be created on the PEEK surface to promote cell infiltration and vascularization, facilitating osseointegration. These structures can be fabricated using techniques such as 3D printing or surface modification methods, as shown in Figure 2. Surface porous PEEK has been shown to promote the proliferation, differentiation, and mineralization of osteoblasts, leading to improved osseointegration [67,73,74]. A study conducted by Wei et al. [75] used magnesium surface-activated 3D-printed porous PEEK scaffolds to enhance the osseointegration capacity of PEEK materials. The surface was coated with polydopamine (PDA) chelated with magnesium ions (Mg^2+^). Following surface modification, bioactive Mg^2+^ was released, and the hydrophilicity of the PEEK scaffolds was greatly increased. The results showed that the customized three-dimensional porous structure facilitated bone ingrowth within the PEEK scaffolds, and the released Mg^2+^ accelerated early bone ingrowth by promoting early angiogenesis during the coating degradation process. Xu et al. [76] developed a Dex/Mino liposome-modified PEEK surface; this surface modification presented favorable stability, cytocompatibility, and improved osseointegration compared to bare PEEK, giving a potential as an orthopedic/dental implant material for clinical application. A recent study proposed a novel surface modification method to obtain a three-dimensional (3D) hierarchical porous structure on the PEEK surface, which showed a boosted osseointegration potential. The hierarchical porous architecture was designed to mimic the natural bone structure and enhance the interaction between the implant and surrounding bone tissue, promoting better osseointegration. More contact areas with the host bone and tissue ingrowth are made possible by the hierarchical topological structure, which results in better mechanical interlocking with the newly created bone tissue [77].

The clinical outcomes and success rates of PEEK dental implants have been evaluated in several studies [78]. A study evaluated the clinical survival and success rate of PEEK composite dental implants using immediate and delayed loading protocols, and the results showed that PEEK composite dental implants had a high clinical survival and success rate (64–76%) [79]. Another study conducted by Ayyadanveettil et al. [30] showed that zirconia and PEEK abutments exhibited the same survival rate with similar biological and esthetic outcomes at the 5-year evaluation. However, there is still a lack of evidence regarding dental implant materials. A longer follow-up period, greater number of patients, and different tooth types (variation in occlusal forces) are required for more accurate results.

### 3.3. PEEK-Based Dental Prosthetics

**Figure 7:**
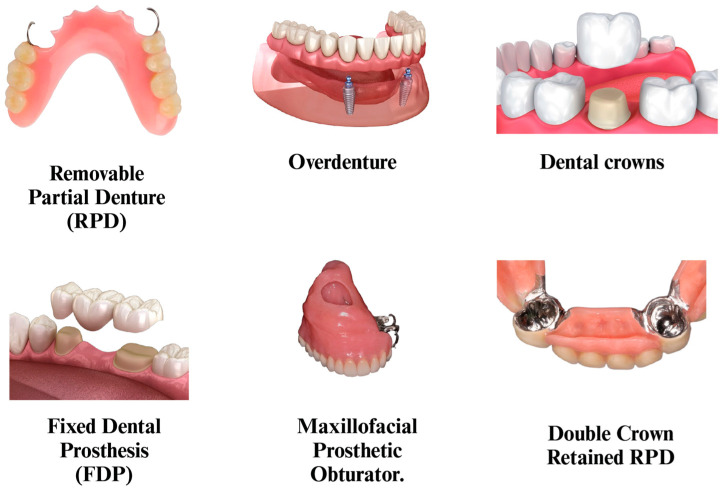
PEEK-based prosthetic restorations.

PEEK has also been explored for various applications in prosthodontics, including crowns, bridges, and dentures [36] (Figure 7). According to a narrative review of CAD-CAM PEEK dental prostheses, PEEK exhibits excellent mechanical characteristics and strong bonding with veneering composite materials, making it an attractive material for fixed dental prostheses. Similar to zirconia and lithium disilicate crowns, PEEK prostheses have been shown to have high fracture resistance and capacity to tolerate occlusal stresses in the molar region. Additionally, PEEK could be considered a material that works effectively for denture bases because it resists fracture and notch concentration [80]. A systematic review of PEEK-fixed partial dentures found that PEEK frameworks showed better esthetics compared to metal frameworks [81]. PEEK biomaterial has been investigated for a number of clinical dentistry applications, such as implant-supported prostheses, fixed dental prostheses, and removable dental prostheses, according to a literature review of PEEK biomaterial in prosthodontics [82]. Fixed Dental Prostheses (FDPs) made from pre-pressed PEEK blanks utilizing CAD-CAM milling showed greater fracture loads. (2354 N) and less deformation than those that were granularly pressed (1738 N). This implies that employing pre-pressed PEEK and CAD-CAM technologies can produce FDPs that are more resilient [83]. PEEK has been shown in other in vitro investigations to be an excellent substitute material for permanent dental prostheses and single crowns. Its potential for load-bearing areas was verified by three-unit PEEK frameworks, which showed FDP connector fracture at 1383 N and deformation at 1200 N [84]. The fracture resistance of CAD-CAM-milled PEEK FDPs is substantially higher than that of zirconia (981–1331 N), alumina (851 N), and lithium disilicate (950 N) when placed against other dental materials [80,83]. This suggests that PEEK (and its composites) may offer superior durability in certain clinical scenarios. When compared to materials like PMMA, composite resin paste, and fiber-reinforced composites, PEEK demonstrated the maximum load-bearing capacity in an in vitro analysis comparing several inlay-retained FDPs [85].

PEEK-based dental prostheses offer a viable alternative to traditional materials owing to their superior biocompatibility, patient acceptance, and advantageous mechanical characteristics. However, further investigation is required to assess their long-term performance and success rates. From a biocompatibility point of view, PEEK exhibits superior biocompatibility, making it suitable for dental applications. Its insolubility in water makes it ideal for use in patients with allergies. PEEK has been found to be non-mutagenic and non-cytotoxic, ensuring its safety for intraoral use [86]. In terms of patient acceptance, PEEK-based dental prostheses have shown good patient acceptance due to their esthetics and metal-free nature. Implant restorations are frequently chosen by patients who have lost their teeth, and PEEK has been suggested for a variety of implant-supported prostheses [8,86]. Regarding mechanical properties, PEEK-based dental prostheses have been found to have high fracture resistance and load-bearing capacity, comparable to other fixed dental prosthesis materials [35]. In regard to bonding issues, bonding between PEEK and resin-based luting materials, including Panavia V5, has been achieved, ensuring the stability and longevity of PEEK-based dental prostheses [87,88,89,90]. PEEK dental prosthesis’ long-term survival cannot be confirmed due to the lack of evidence [81]. However, some studies have reported high clinical survival and success rates of PEEK-based dental prostheses for up to 5–8 years [81,91]. To assess the long-term effectiveness and success rates of dental prostheses made from PEEK, additional studies are required [92]. PEEK-based dental prostheses require regular maintenance, including cleaning and polishing, to prevent plaque buildup and staining. PEEK is a highly durable material that can withstand mechanical and physical stress, which makes it a perfect material for dental implants and devices that require long-term durability. However, regular maintenance is still necessary to ensure the longevity of PEEK-based dental prostheses [86]. PEEK-based dental prostheses can be repaired using composite resin materials that bond well with PEEK. However, the repairability of PEEK-based dental prostheses may depend on the extent of damage and location of the prosthesis. PEEK-based dental prostheses may need to be replaced over time due to wear and tear or damage. Replacement may be necessary if the prosthesis becomes loose or damaged beyond repair [93].

A one year single-arm pilot study evaluated the clinical acceptability of PEEK-fixed dental prostheses in partially edentulous patients. The study found that PEEK-fixed dental prostheses were clinically acceptable, but further research is needed to evaluate their long-term survival and success rates [94]. Another study evaluated the survival rates and treatment success of full-arch implant-supported PEEK prostheses. The study found that the dental implant survival rate was 99% for up to 77 months (6 years and 5 months) and the PEEK prosthesis survival rate was 100% for up to 56 months (4 years and 8 months). Bone loss after an average of 54 months (4 years and 6 months) was minimal [92]. However, systematic reviews of clinical studies have found inadequate data to determine the long-term survival of PEEK dental prostheses. Another interesting study evaluated the clinical results of edentulous patients receiving implant-supported fixed complete dentures with PEEK CAD-CAM frameworks compared to titanium frameworks. The study found that PEEK CAD-CAM frameworks were a viable alternative to titanium frameworks, with a high clinical success rate (100%) [95,96].

In terms of practical applications, a more recent systematic exploration by Mishra et al. [97] highlighted that complete-arch implant-supported fixed dental prostheses fabricated with PEEK exhibited a cumulative survival rate of 97.3% over a follow-up period ranging from 1 to 6 years, indicating promising initial outcomes. Their analysis was informed by 12 clinical studies, with a total of 119 prostheses assessed, underscoring feasibility in clinical settings. While the performance metrics appear encouraging for the short-term, the author’s cautionary note about adhesion issues indicates an area requiring additional rigorous inquiry. A noteworthy consideration highlighted by Qin et al. [98] is the treatment of the PEEK surface, which is intrinsically hydrophobic. This characteristic has obstructed its bonding efficacy with dental resin composites, presenting a barrier to its widespread use despite the favorable physical and mechanical properties identified in the literature. Innovative surface modifications are posited as crucial pathways toward enhancing the clinical applicability of PEEK-based systems, aligning with the contemporary focus on improving biomaterials in dentistry. Alexakou et al. [99] reported that despite the promising direction of PEEK as a dental restorative material, the clinical translation remains limited by the scarcity of definitive longitudinal studies that track patient outcomes over an extended timeframe.

**Figure 8:**
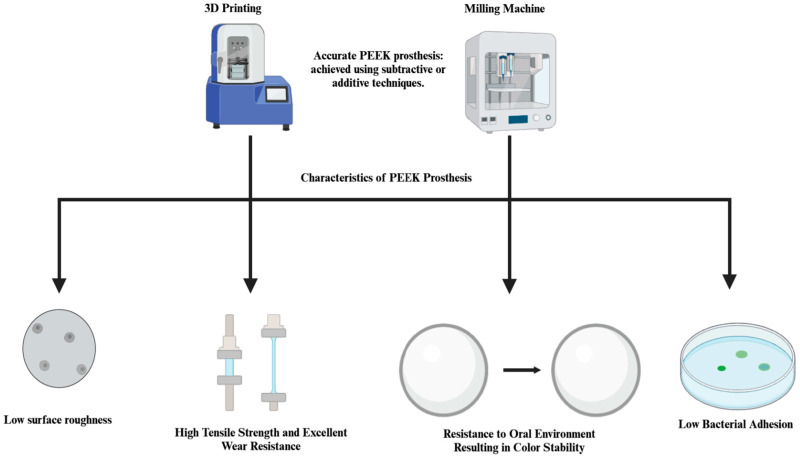
Significant material properties of 3D printed vs. CAD/CAM-milled PEEK prostheses.

PEEK’s surface characteristics play a significant role in its polishability and subsequent bacterial adhesion, which is critical for prosthetic applications. It demonstrates good polishing properties, which are important for reducing bacterial adhesion and plaque accumulation. PEEK surfaces can be polished to achieve a roughness (Ra) below 0.13 µm. This level of smoothness is considered favorable for reducing bacterial adhesion [100]. Glazing of PEEK surfaces has a positive effect on surface roughness, regardless of artificial aging or staining protocols [101]. Surface irregularities such as flaws and defects promote bacterial attachment by providing protection against shear forces [100]. Pressed PEEK showed higher bacterial adhesion compared to milled, injection-molded, or 3D-printed PEEK. Horizontally, 3D-printed PEEK exhibited lower bacterial adhesion compared to vertically printed specimens. Surface roughness below 10 µm or additional polishing appears to be essential for reducing bacterial adhesion on PEEK surfaces [102] (Figure 8).

### 3.4. PEEK in Bone Regeneration

#### 3.4.1. PEEK in Alveolar Ridge Preservation and Augmentation

Alveolar ridge augmentation provides sufficient and precise regenerated bone tissue for subsequent dental implant placement [103]. A comparative study based on 3D printing technology found that customized alveolar bone augmentation using PEEK scaffolds is a viable alternative to titanium scaffolds [104,105,106]. PEEK has been compared to other materials, including titanium mesh, for use as a bone replacement material. PEEK has a higher cost compared to titanium mesh. However, the cost of the combination of titanium mesh and bone cement is reportedly similar to that of PEEK if surgical time and other factors are considered [107]. PEEK exhibits good biocompatibility and radiolucency, making it a potential material for use in cranioplasty and other medical applications. Titanium mesh is commonly used for cranioplasty, but PEEK has been found to have a lower complication rate and implant failure rate compared to titanium mesh [108]. PEEK has a lower elastic modulus than titanium, making it more suitable for craniofacial and orbital floor defects [109]. In comparison to titanium grade 2 and titanium grade 5, PEEK exhibits greater bacterial adhesion [110,111].

#### 3.4.2. Guided Bone Regeneration with PEEK

In order to heal defects in the bone and rebuild the alveolar ridge, PEEK membranes have been examined for use in guided bone regeneration (GBR). A membrane is utilized in GBR to separate soft tissues and boost bone regeneration. The success of GBR is largely dependent on the implementation of barrier membranes. PEEK membranes have been proposed for GBR due to their antibacterial and bone-binding capabilities. However, the economic cost, clinical feasibility, and processing complexity of PEEK modification remain to be evaluated [112,113,114]. PEEK can be modified to possess antibacterial properties that can reduce the risk of infection and promote bone regeneration. The antibacterial properties of PEEK can be achieved through surface modification or blending modification [115,116,117]. PEEK has been found to have good bone-binding capabilities, making it a potential material for GBR. PEEK can be modified to enhance its hydrophilicity and protein adsorption capacity, thereby promoting osteogenesis [27,118,119]. PEEK offers a stable environment for bone regeneration and can be utilized as a tailored barrier membrane for GBR. PEEK membranes have been found to have good integration behavior and low immune response, making them a suitable material for GBR. Severe atrophic alveolar bones can be effectively supported using customized PEEK membranes [112,114,120]. However, the practical use of PEEK is restricted due to its poor capacity for osteogenesis and osseointegration. The clinical viability, economic cost, and processing complexity of PEEK modification remain to be evaluated. The antibacterial and bone-binding capabilities of PEEK can be enhanced through surface modification or blending modification. Further research is needed to evaluate the clinical feasibility and long-term performance of PEEK membranes for GBR compared to other materials [112].

#### 3.4.3. PEEK-Based Scaffolds for Tissue Engineering

Cells, scaffolds, and growth factors are used in tissue engineering to replace or regenerate diseased or damaged tissues. The mechanical, biodegradable, and biocompatible characteristics of polymeric scaffolds have made them popular for tissue engineering applications. PEEK scaffolds have been found to have good mechanical properties, including high fracture resistance and load-bearing capacity, making them suitable materials for tissue engineering [75,121,122].

Because of their unique features, such as their high mechanical strength, radiolucency, and good biocompatibility, tissue engineering has made use of PEEK scaffolds. Tissue healing is facilitated by scaffolds, which enhance the differentiation of cells and proliferation. PEEK scaffolds have been found to promote osteogenic bone regeneration and delay adjacent bone loss. PEEK scaffolds have also been used in bone tissue engineering to provide a 3-dimensional environment for cell seeding and proliferation, as well as filling bone defects [11,121,123]. The integration of stem cells and growth factors into PEEK scaffolds has been explored in regenerative medicine and tissue engineering [124]. Scaffolds made of PEEK may contain growth factors for extended release, which promotes osteogenesis. However, mismatched release profiles, in which the growth factor release is frequently unsteady, are typically linked to difficulties with growth factor-enriched scaffolds [125]. Stem cells can be seeded onto PEEK scaffolds to promote bone regeneration and tissue healing. Roskeis et al. [126] used a 3D-printed scaffold embedded with mesenchymal stem cells in an effort to increase PEEK bioactivity for craniofacial repair. Computer-aided design software was used to create customized PEEK scaffolds with a trabecular microstructure, which were subsequently printed using selective laser sintering (SLS). The scaffold structure was evaluated using micro-CT, and SEM was used to evaluate scaffold morphology with and without mesenchymal stem cells (MSCs). The study found that PEEK scaffolds maintained the viability of both adipose-derived stem cells (ADSCs) and bone marrow-derived mesenchymal stem cells (BMSCs); however, ADSCs demonstrated higher osteodifferentiation than BMSCs. Another study evaluated the influence of porous silk scaffolds on the delivery of growth factors and stem cells to enhance bone regeneration. PEEK scaffolds can also be used to seed cells that have been altered to express osteoinductive growth factors, producing a comparable result. Gene therapy carried out by viral or nonviral transduction is usually required to modify the cells [127]. When evaluating how human mesenchymal stem cells (hMSCs) function following their seeding on extremely porous trabecular titanium (T-Ti) or PEEK, the study showed that when it comes to the manner in which human adipose stem cells differentiate into cells that generate bone in 3D culture, osteogenic media outperform growth factors. While hMSCs cultured on the T-Ti scaffold demonstrated uniform colonization of the scaffold, those cultured on PEEK in osteogenic media showed a decrease in cellular density [128].

## 4. Clinical Outcomes and Success Rate of PEEK Materials

The clinical outcomes and success rates of PEEK materials are promising, with high survival rates reported for PEEK implants, PEEK crowns, PEEK abutments, and PEEK bridges in several studies. However, more long-term studies are needed to ascertain the long-term survival and clinical acceptability of PEEK materials.

Siewert et al. [129] reported positive long-term survival rates and patient satisfaction with PEEK implant-supported full-arch prostheses. Montero et al. [130] found that PEEK rehabilitation significantly improves bite force, occlusal arrangement, masticatory function, patient satisfaction, and quality of life. PEEK was shown by Kwan et al. [131] to be a suitable temporary fixed dental prosthesis during implant treatment, with good results and limited complications. However, Gowda et al. [79] highlighted a higher failure rate for immediately loaded PEEK implants compared to those loaded using an early loading protocol. Most research suggests that PEEK dental materials can offer favorable clinical outcomes and patient satisfaction; however, careful consideration of loading protocols may be necessary for optimal success rates.

Wang et al. conducted a comparative study of PEEK and titanium overdentures. The 5-year survival rates were comparable, with those of PEEK at 93.1% and titanium at 93.5%. PEEK showed slightly better outcomes in terms of peri-implant health: bleeding on probing: PEEK 13.8% vs. titanium 16.1%, soft tissue inflammation: PEEK 3.4% vs. titanium 3.2%, vertical bone loss: PEEK 0.70 mm vs. titanium 0.96 mm [8].

Tasopoulos et al. reported a case study of a 47-year-old patient who received a two-piece PEEK maxillary obturator. After one year of follow-up, no complications were reported, suggesting promising outcomes for PEEK in maxillofacial applications [132].

While these studies demonstrate promising outcomes for PEEK in dental applications, it is important to note that long-term data are still limited. A systematic review protocol conducted in (2024) aims to comprehensively assess the clinical efficacy of PEEK in the all-on-four concept, which may provide more definitive evidence in the future [133]. In addition, Khurshid et al. reported that the majority of the evidence regarding the outcomes of PEEK dental prostheses is obtained from case reports and non-randomized observational studies [81].

Although long-term data on PEEK prosthetics in clinical use are still growing, early results from follow-ups highlight their durability, reduced failure rates, and lower complications, such as mechanical fractures or material wear, compared to traditional prosthetic materials.

## 5. Future Directions and Emerging Trends

One of the emerging trends is the customization of PEEK dental prosthetics. Advanced technologies like 3D printing and CAD/CAM (computer-aided design and computer-aided manufacturing) are being used to create patient-specific dental implants, crowns, and bridges. This approach can enhance the fit and aesthetics of dental prostheses [15,134,135]. 3D printing technology has been explored for the fabrication of dental prostheses, including crowns, bridges, and dentures [136]. 3D printing technology has also been explored for the fabrication of customized periodontal scaffolds for the regeneration of oral soft tissues [137]. The integration of PEEK-based dental materials into digital workflow and teledentistry could streamline treatment planning and delivery, enabling more efficient and patient-centered care. Interest in the development of hybrid materials that combine PEEK with other substances like bioceramics or bioactive agents to leverage PEEK’s mechanical properties while enhancing its biological interactions is growing rapidly. Moreover, advanced techniques such as plasma treatment and coating with bioactive materials can potentially enhance PEEK’s bioactivity. Continued research on implant design, including macrostructure and microstructure modifications, can lead to more successful and durable PEEK dental implants [42]. These improvements may enhance stability and reduce the risk of implant failure. The use of PEEK-based dental materials and 3D printing technology in dentistry raises regulatory considerations. The safety and efficacy of PEEK-based dental materials and 3D printing technology need to be evaluated through clinical trials and regulatory approval processes.

## 6. Conclusions

PEEK is a highly promising material in the field of dentistry, with a wide range of applications across various specialties. Key findings from the existing literature highlight PEEK’s exceptional mechanical properties and its versatility in removable and fixed dental prostheses. Researchers have also been actively exploring ways to enhance the bioactivity of PEEK implants, opening up new avenues for implantology. PEEK’s notable qualities, including mechanical strength, biocompatibility, chemical stability, and radiolucency, position it as an ideal material for removable dental prostheses. Furthermore, its applications extend to implantology, removable denture frameworks, fixed partial dentures, and orthodontic wires. Moreover, PEEK’s unique properties have paved the way for groundbreaking developments in tissue engineering and regenerative medicine, particularly in areas such as bone regeneration and the creation of customized periodontal scaffolds. By leveraging 3D printing technology, dental professionals have gained greater flexibility in designing and fabricating dental prostheses and scaffolds, thereby ushering in a new era of personalized dentistry. However, it is imperative to stress that clinical trials and regulatory approval procedures should be undertaken to thoroughly assess the safety and effectiveness of PEEK-based dental materials and 3D printing technologies. This step is vital to ensure that these innovations meet the highest standards and adhere to ethical considerations.

In summary, PEEK-based dental materials hold significant promise for the future of dentistry, offering a unique combination of properties and the potential for personalized treatment approaches. However, ongoing research is essential to assess their clinical feasibility and long-term performance and navigate the complex landscape of regulatory and ethical considerations, ultimately working toward improved patient outcomes in the field of dentistry.

## Figures and Tables

**Table 1:** Properties of PEEK compared to those of other polymers.

Material	Specific Gravity (g/cm^3^)	Glass Transition Temperature (°C)	Melting Point (°C)	Tensile Strength (MPa)	Young Modulus (GPa)	Operating Temperature (°C)	Elongation at Break (In %)	Ref.
PEEK	1.3	143	340	86–94	3–4	250	-	[4,42]
PEKK ^1^	1.27	162	305	105	5.1	300	-	[4]
UHMWPE ^2^	0.933	−110	200–220	48	0.69	<100	-	[4]
PLLA ^3^	-	51–64	172–189	27.98–49	-	-	5	[43]
DL-PLA ^4^	-	50–52	Amorphous	28.9–35	-	-	6	[43]
Tendon Chitosan	-	-		56.48–79.21	-	-	11.3–13.92	[43]

^1^ Polyetherketoneketone. ^2^ Ultra-high molecular weight polyethylene. ^3^ Poly-l-lactic Acid. ^4^ Poly(dl-lactic acid).

**Table 2:** PEEK properties compared to bone and other dental materials.

Material	Young’s Modulus (GPa)	Tensile Strength (MPa)	Flexural Strength (MPa)	Hardness	Degradability	Ref.
Cancellous Bone	2–4	13–17	12–18	0.46 ± 0.08	Biodegradable	[42,57]
Cortical Bone	27–33	105–115	118–122	0.43 ± 0.13	Biodegradable	[42,57]
PEEK	3–4	86–94	110–120	85–109	Nonbiodegradable	[42,57]
20 wt.% CFR-PEEK	18–20	125–131	160–168	-	Nonbiodegradable	[42]
30 wt.% CFR-PEEK	22–28	151–157	163–173	-	Nonbiodegradable	[42]
GFR-PEEK	10–12	115–158	198–228	-	Nonbiodegradable	[42]
Zirconia	200–10	320–340	240–260	-	Nonbiodegradable	[42]
Titanium alloy	110–119	862–1200	-	337–357	Nonbiodegradable	[57]

**Table 3:** Mean compression elastic modulus and flexural strength of PEEK nanocomposites with respect to the proportions of bioglass (45S5 bioglass) and forsterite (Mg_2_SiO_4_) nanofillers. PKBG 10, PKBG 20, and PKBG 30 are PEEK containing 10, 20, and 30 wt. %, bioglass nanofillers, respectively, and PKFT 10, PKFT 20, PKFT 30 is PEEK containing 10, 20, 30 wt.% Forsterite nanofillers, respectively [6].

PEEK Nanocomposites	Elastic Modulus (MPa) Mean ± SD	Flexural Strength (GPa) Mean ± SD
Pure PEEK	3.8 ± 0.13	143.63 ± 3.26
PKBG 10	4.19 ± 0.13	149.43 ± 6.11
PKBG 20	4.63 ± 0.12	151.3 ± 6.69
PKBG 30	3.64 ± 0.14	121.2 ± 6.4
PKFT 10	4.4 ± 0.14	166.33 ± 9.2
PKFT 20	6.02 ± 0.11	200.19 ± 4.12
PKFT 30	4.98 ± 0.17	188.32 ± 4.13

## Data Availability

All data analyzed in this study are included in this published article.
